# Regulation of cardiac function by cAMP nanodomains

**DOI:** 10.1042/BSR20220953

**Published:** 2023-02-27

**Authors:** Milda Folkmanaite, Manuela Zaccolo

**Affiliations:** Department of Physiology, Anatomy and Genetics, University of Oxford, U.K.

**Keywords:** cAMP, cardiovascular disease, compartmentalization, intracellular signaling, nanodomain, personalized medicine

## Abstract

Cyclic adenosine monophosphate (cAMP) is a diffusible intracellular second messenger that plays a key role in the regulation of cardiac function. In response to the release of catecholamines from sympathetic terminals, cAMP modulates heart rate and the strength of contraction and ease of relaxation of each heartbeat. At the same time, cAMP is involved in the response to a multitude of other hormones and neurotransmitters. A sophisticated network of regulatory mechanisms controls the temporal and spatial propagation of cAMP, resulting in the generation of signaling nanodomains that enable the second messenger to match each extracellular stimulus with the appropriate cellular response. Multiple proteins contribute to this spatiotemporal regulation, including the cAMP-hydrolyzing phosphodiesterases (PDEs). By breaking down cAMP to a different extent at different locations, these enzymes generate subcellular cAMP gradients. As a result, only a subset of the downstream effectors is activated and a specific response is executed. Dysregulation of cAMP compartmentalization has been observed in cardiovascular diseases, highlighting the importance of appropriate control of local cAMP signaling. Current research is unveiling the molecular organization underpinning cAMP compartmentalization, providing original insight into the physiology of cardiac myocytes and the alteration associated with disease, with the potential to uncover novel therapeutic targets. Here, we present an overview of the mechanisms that are currently understood to be involved in generating cAMP nanodomains and we highlight the questions that remain to be answered.

Properly executed cardiac contraction and relaxation are essential for maintaining the normal function of the heart. Sympathetic stimulation and catecholamines release prompted under stress conditions (e.g. during physical or emotional strain) modulate cardiac activity via G-protein coupled receptor (GPCR) signaling. Catecholamines activate β adrenergic receptors (βAR), a subfamily of GPCRs, leading to synthesis of cyclic adenosine monophosphate (cAMP) by adenylyl cyclases (AC) and modulation of heart rate (chronotropy), relaxation (lusitropy), and contraction force (inotropy). The signal is then terminated by phosphodiesterases (PDEs) that degrade cAMP to adenosine monophosphate [1–3]. Different types of GPCRs elicit distinct effects: activation of G_s_-coupled receptors leads to increased AC activity, whereas activation of G_i_-coupled receptors inhibits cAMP synthesis by AC [4,5]. Each individual cell can express up to hundreds of different G_s_- or G_i_-coupled receptors [6] that, in response to a variety of hormones and other extracellular signals, lead to changes in cAMP. Each receptor elicits a response that is appropriate to the specific activating extracellular stimulus, despite all these receptors operating via changes in the level of the same second messenger, cAMP.

## cAMP pathway compartmentation

### Compartmentation of protein components

**Figure 1:**
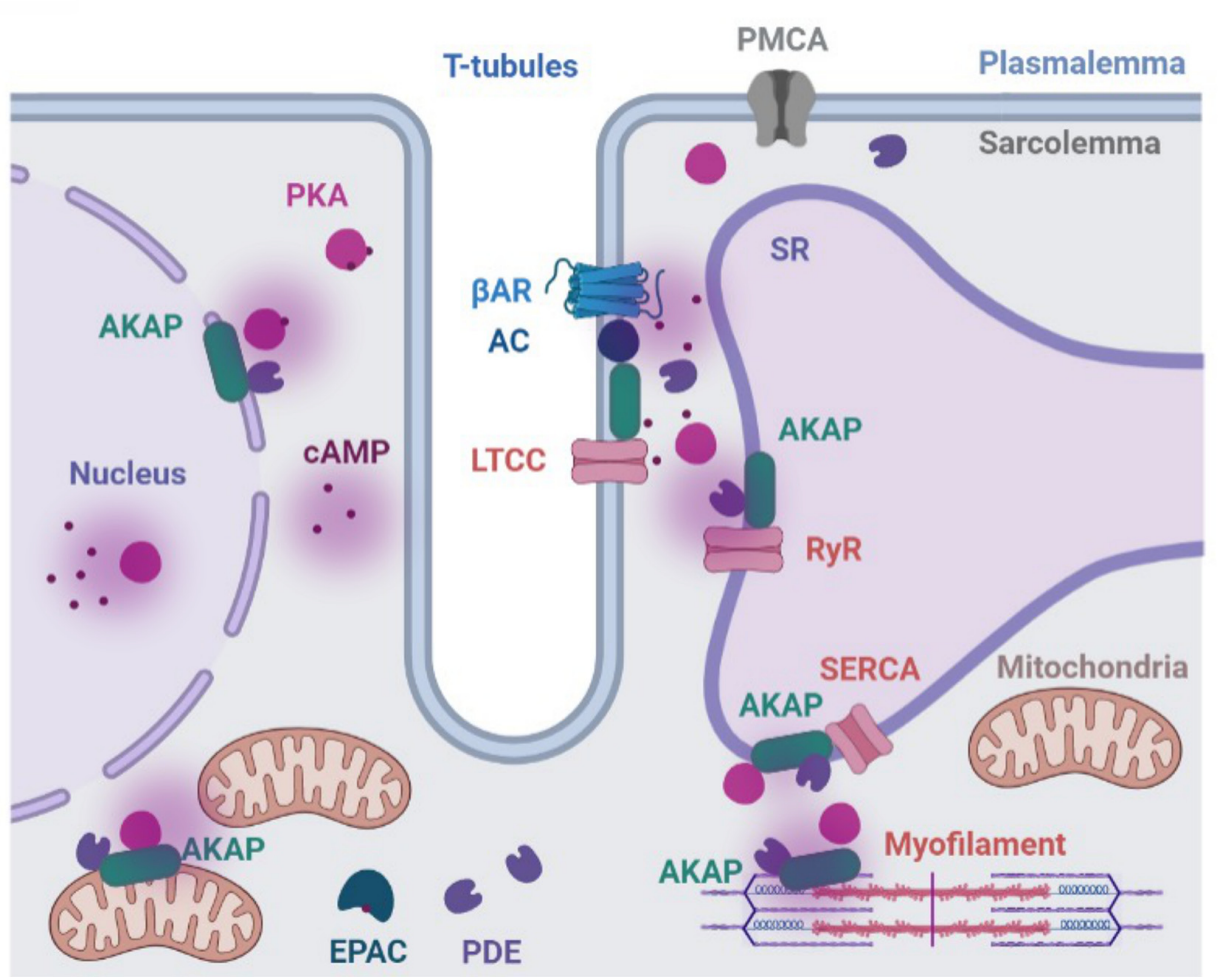
Different cAMP nanodomains execute βAR signaling in cardiomyocytes When βARs are activated, ACs produce cAMP, which diffuses throughout the cytosol. Different cAMP nanodomains help to ensure cAMP signaling specificity. PDEs and AKAPs are two important components of cAMP nanodomains. PDEs degrade cAMP, helping to form cAMP gradients within the cell. AKAPs anchor PKA, a key cAMP effector protein, to specific locations within the cell. Both PDEs and AKAPs have multiple isoforms with varying enzymatic activity, localization, and expression levels. The presence of multiple cAMP signaling components helps ensure that the correct cellular effects are achieved upon βAR activation. Abbrreviations: AC, adenylyl cyclase; AKAP: A-kinase anchoring protein; βAR, β adrenergic receptor; EPAC, exchange protein directly activated by cAMP; PDE, phosphodiesterase; PKA, protein kinase A; PMCA, plasma membrane calcium ATPase; LTCC, L-type calcium channel; RyR, Ryanodine channel; SERCA, SR calcium ATP-ase, SR, sarcoplasmic reticulum.

cAMP, a ubiquitous second messenger produced in response to the GPCR signaling, modulates cardiac contraction and relaxation, as well as multiple other biological responses such as gene expression and regulation of metabolic pathways [7]. cAMP can act through four known effector protein groups: protein kinase A (PKA), exchange protein-activated by cAMP (EPAC), cyclic nucleotide-gated channels (CNGC), and Popeye domain-containing (POPDC) proteins. To enable hormonal specificity, cAMP signals and the activity of its effectors are compartmentalized in the cell (Figure 1) [8–12]. The spatial and temporal regulation of cAMP signaling was first hypothesized when it was observed that stimulation of GPCR with isoproterenol, a βAR activator, or with prostaglandin induces different effects despite generating similar levels of cAMP: βAR stimulation promotes cardiac inotropy and lusitropy while activation of the prostaglandin receptor does not [13]. Such hormonal specificity was then shown to rely on βAR stimulation predominantly activating a subset of type II isoforms of PKA, while prostaglandin mostly activates type I PKA [14]. The heart expresses different types of βAR, including β1, β2, and β3AR that induce distinct effects. For example, activation of β1AR, a predominant isoform in the heart, increases heart rate and cardiac contractility, but its chronic stimulation can lead to cardiac remodeling and heart failure. β2AR signaling also regulates rate and contractility but it can also activate nonclassical signaling pathways, and its effects are more localized than β1 and its activation is cardioprotective [15]. β3AR receptors appear to mediate negative inotropic effects in mice cardiomyocytes [16,17]. Different PKA types are anchored to distinct PKA-anchoring proteins (AKAPs). Selective activation of restricted subsets of anchored PKA is then achieved via generation of subcellular cAMP gradients, resulting in phosphorylation of different protein targets. Several other studies provide additional support to spatial confinement being essential for achieving hormonal signaling specificity and accurate execution of cellular functions [11,18–22].

Multiple factors contribute to signal compartmentalization, including distinctive signaling via multiple GPCRs, involvement of different PDE isoforms, AKAPs, phosphatases (PP), ACs, and types of effector proteins such as PKA and EPAC. For example, activation of β1-ARs, but not β2-ARs, leads to phosphorylation of phospholamban (PLN) [23,24]. This can be attributed to differential localization of the receptors, as β2-ARs localize predominantly to the T tubules, whereas β1-ARs can be found across the entire cell membrane and generate a more diffuse cAMP response. Signaling through distinct AC isoforms also elicits specific effects, likely due to differential localization of AC isoforms. For example, disruption of AC5 signaling has been demonstrated to be cardioprotective, whereas AC6 knockout results in increased mortality upon β-AR stimulation-induced cardiomyopathy [25,26]. AKAPs ensure signaling specificity by anchoring PKA to intracellular sites, thereby confining phosphorylation signals to specific targets. AKAPs promote signaling specificity also by binding a different array of cAMP signaling-associated proteins, thus generating localized signaling hubs, or signalosomes. The association of the various signaling components with the AKAP may have different effects. For example, specific AKAP-AC interactions promote cAMP generation and PKA signaling, whereas AKAP interaction with other AC inhibits the cyclase activity [27]. Overall, specific protein–protein interactions involving pathway components, in combination with mechanisms of restricted diffusion and buffering of cAMP, contribute to the formation of local cAMP gradients and signal compartmentation [28].

PDEs, the cAMP-degrading enzymes, play a key role in the control of cAMP compartmentation. PDEs belong to a superfamily of enzymes including 11 families (PDE1–11) and over 100 estimated isoforms [29]. Different PDE isoforms have distinct localization, allosteric modulation, substrate specificity (for cAMP and/or cGMP) and enzymatic kinetics. These different features contribute to the ability of individual PDE isoforms, or a specific combination of PDE isoforms, to establish cAMP nanodomains where the local cAMP concentration is differentially regulated from neighboring domains. Investigations on the role of PDEs on cAMP signaling in the heart suggest that PDE control might not be static, but rather change depending on activation of different signaling pathways. For example, it appears that PDE4 inhibition does not influence troponin I (TnI) phosphorylation in resting rat cardiac myocytes, while PDE4 is reportedly the main PDE that regulates cAMP levels at the TnI cAMP nanodomain upon βAR stimulation [30]. It is unclear, however, if these results translate to human as PDE4 expression is significantly lower in human than in rodent hearts [31].

A significant advancement in our understanding of cAMP regulation in the heart has been enabled by the use of genetically encoded fluorescence energy transfer (FRET)-based biosensors. Targeting these sensors to subcellular sites and acquiring live cell, real-time data with high spatiotemporal resolution have allowed direct observation of cAMP compartmentation and dissection of important details of signal regulation. Targeted FRET sensors have been useful in highlighting the importance of PDEs in forming distinct subcellular cAMP-signaling domains. For example, cAMP FRET sensors targeted to different subcellular sites revealed that cAMP levels following βAR stimulation are significantly lower at the myofilament than at other sites, including the sarcoplasmic reticulum (SR) and the plasmalemma in rat cardiomyocytes [32]. Interestingly, the differences in local signaling were abolished after PDE inhibition, highlighting the importance of PDE in the control of local cAMP signals. Several other studies report the central role of PDEs in establishing cAMP compartmentation [33–35]. FRET sensors have also been instrumental in determining the size of the cAMP-signaling domains elicited by different PDEs. Using FRET sensors where a linker of varying length was introduced between the sensor and the catalytic domain of a PDEs, it was shown that different PDEs form cAMP domains of different sizes, e.g. the catalytic domain of PDE2A3 enzymes created a 30-nM nanodomain with low cAMP, whereas the catalytic domain of PDE4A1 generated a domain less than 10 nm in diameter in HEK293 cells [36]. A recent study used a similar approach to demonstrate that activation of GLP-1 receptors generates a pool of high cAMP that extends up to 60 nm in diameter around the receptor [37]. Spatially restricted cAMP generation upon GPCR simulation would permit precise activation of downstream pathways and signaling cascades that are associated specifically with that specific receptor, avoiding cross-activation. Domains with similar nanometer size were observed at different subcellular sites including myofilament, SR, and sarcolemma [28,32,38].

### Compartmentation of cAMP

For the cell to be able to manage locally controlled cAMP nanodomains, the production, degradation, and propagation of cAMP must be precisely regulated. How this is executed remains to be fully elucidated. Estimates of intracellular cAMP diffusion coefficient range from 10 to 780 μm^2^/s, depending on cell type and experimental conditions [39,40]. Using experimentally established values, computational diffusion models indicate that PDEs alone cannot explain cAMP compartmentation as PDEs have relatively low catalytic rates that seem inadequate to effectively restrict the highly diffusible cAMP [40]. Even when taking into account that the activation threshold of PKA *in vivo* may be more than 20-fold higher than previously established *in vitro* [41], the activity of the PDEs alone would still appear inadequate to create functionally meaningful cAMP gradients that can selectively activate only a subset of anchored PKA. This view could change if the PDE enzyme kinetics in the cell were also significantly different from those measured *in vitro*, as described for PKA. However, this remains to be established.

Mathematical models that integrate our current knowledge on PDE activity suggest that these enzymes could generate gradients of cAMP if the diffusion coefficient of the second messenger was significantly reduced [40]. Factors that could slow down cAMP diffusion in the cell include physical barriers and cAMP buffering. The expression of cAMP-binding proteins has traditionally been thought to be limited, making cAMP buffering an unlikely mechanism to influence cAMP dynamics. However, a significant molar excess of PKA regulatory subunits compared with catalytic subunits (average ∼17-fold) has been reported, which could potentially provide substantial cAMP buffering [42].

In support of significant intracellular-buffering capacity, a recent study reports that, at low concentrations, a fluorescent cAMP analog diffuses extremely slowly at 0–10 μm^2^/s within the cell while its diffusivity increases to 10–100 μm^2^/s [36] when the cAMP concentration is increased by AC activation and PDE inhibition in HEK293 cells. Lack of diffusion at low cAMP concentration suggests that a number of cellular cAMP-binding sites exist that may enable the formation of cAMP nanodomains. Hydrolysis of cAMP by PDEs, together with intracellular cAMP buffering, could explain how cAMP signaling is compartmentalized in cells, although further investigations are warranted to determine the amount of non-PDE-based buffering needed for the cell to be able to maintain cAMP nanodomains.

A novel mechanism for how cAMP buffering could be executed in the cell has been recently reported by Zhang et al. The study shows that PKA regulatory subunits type Iα (RIα) undergoes liquid–liquid phase separation (LLPS) in the cytosol to form biomolecular condensates in various cell types, including cardiomyocytes [43]. Protein LLPS is a process where proteins self-assemble into liquid droplets that function as membraneless organelles, enabling the cell to optimize reaction kinetics, buffer molecules, sense stress, and localize signals, among other functions [44]. Via LLPS, PKA RIα forms membraneless organelles that contain cAMP at a concentration that is higher than the cAMP concentration in the bulk cytosol. These organelles are thought to provide a cAMP-buffering function, and might also include other molecules involved in cAMP signaling such as ACs, PDEs, or other, although direct evidence is still lacking. Fluorescence recovery after photobleaching (FRAP) experiments performed using fluorescent cAMP show a slower fluorescence recovery within RIα condensates versus cAMP in the surrounding cytosol, with the diffusivity being around 0.004 µm^2^/s for cAMP in the droplets and more than 100 µm^2^/s for the regions outside the droplets. Based on these observations, the authors conclude that RIα condensates might be able to act as a buffer for cAMP. The binding of cAMP to RIα activates the catalytic (C) subunit, allowing it to phosphorylate its targets [45]. RIα condensates appear to encompass also C subunits, providing a model for how cAMP, and C subunits, can be entrapped locally thus contributing to local regulation of signaling [43].

These exciting new findings prompt further questions regarding what the function of the RIα condensates might be in the cell besides their proposed buffering effect. Other biomolecular condensates are known to be important for promoting enzymatic reaction optimizations, but it is currently unknown if and how LLPS affects PKA activity [44]. Computational approaches integrating cAMP diffusion and PKA measurements with PKA LLPS could help elucidate the required ratios of diffuse to condensed PKA for buffering to occur. Other cAMP-binding proteins, in principle, could provide a similar cAMP-buffering mechanism, if they were able to phase-separate. Indeed, a recently published study suggests that cAMP can act through EPAC1 to trigger LLPS and form nuclear condensates [46]. EPAC1 seems to be able to form condensates *in vitro* and in cells, and the concentration of the protein required for condensation *in vitro* is reduced upon cAMP addition.

The suggestion that cAMP can directly modulate the dynamics of biomolecular condensates including PKA RIα and EPAC1 is interesting as this would provide a mechanism for regulation of protein-phase separation by an endogenous ligand. It remains unclear if cAMP diffusion coefficient inside the nuclear EPAC1 condensates is reduced in a similar way as reported for RIα puncta [43]. If confirmed, the ability of cAMP to directly regulate the dynamics of phase separation would provide another important mechanism to achieve compartmentalized signaling in basal and stress conditions. Further investigations of the functional impact of PKA-phase separation will hopefully help elucidate its physiological roles.

## Regulation of cardiac function by cAMP nanodomains

In the heart cAMP-mediated signaling regulates calcium handling, myofilament function, gene expression, and metabolism. The small size of cAMP nanodomains allows specific regulation, preventing signaling interference that may derive from modulation of proteins that are in relative vicinity to each other but are tasked with executing distinct cellular effects. One example is the generation of distinct cAMP pools by β-AR and GLP-1 receptors at the plasmalemma mentioned above. This could in principle apply also to L-type calcium channels (LTCC) and Ryanodine receptors (RyR) in the dyadic cleft, or troponin and titin at the myofilament. The tight spatial regulation of cAMP nanodomains provides an opportunity for targeted therapeutic intervention and the potential for development of more effective drugs with reduced side effects. For example, the PDE3 inhibitor milrinone has an acute positive inotropic effect and enhances stroke volume in HF patients [47]. However, in the long term, PDE3 inhibition increases the likelihood of arrhythmias and its administration to patients is associated with increased mortality [48,49], limiting its indication to the treatment of acute, refractory HF. Such side effects could perhaps be avoided by targeting more selectively one of the four PDE3 isoforms rather than inhibiting global PDE3 activity [50]. An approach that disrupts protein–protein interactions between AKAP and PKA or AKAP and a PDE might prove to be more successful, as it would allow to modulate cAMP levels very selectively at an individual signalosome. Below, we review the current knowledge on how cAMP nanodomains are involved in the regulation of cardiac function, how they impact cardiovascular diseases and how they could potentially be targeted for therapeutic purposes.

### Regulation of calcium handling

cAMP regulation of calcium signaling at subcellular sites ensures proper cardiac function [51–53]. PKA-dependent phosphorylation regulates the function of some of the main players in the regulation of the calcium transient that drives contraction and relaxation, including the activity of LTCCs and, via PLN phosphorylation, SR calcium reuptake. In disease, impaired calcium cycling induces abnormal cardiac contractility leading to cardiovascular disorders including HF and arrhythmias [54–56].

#### Domains

**Figure 2:**
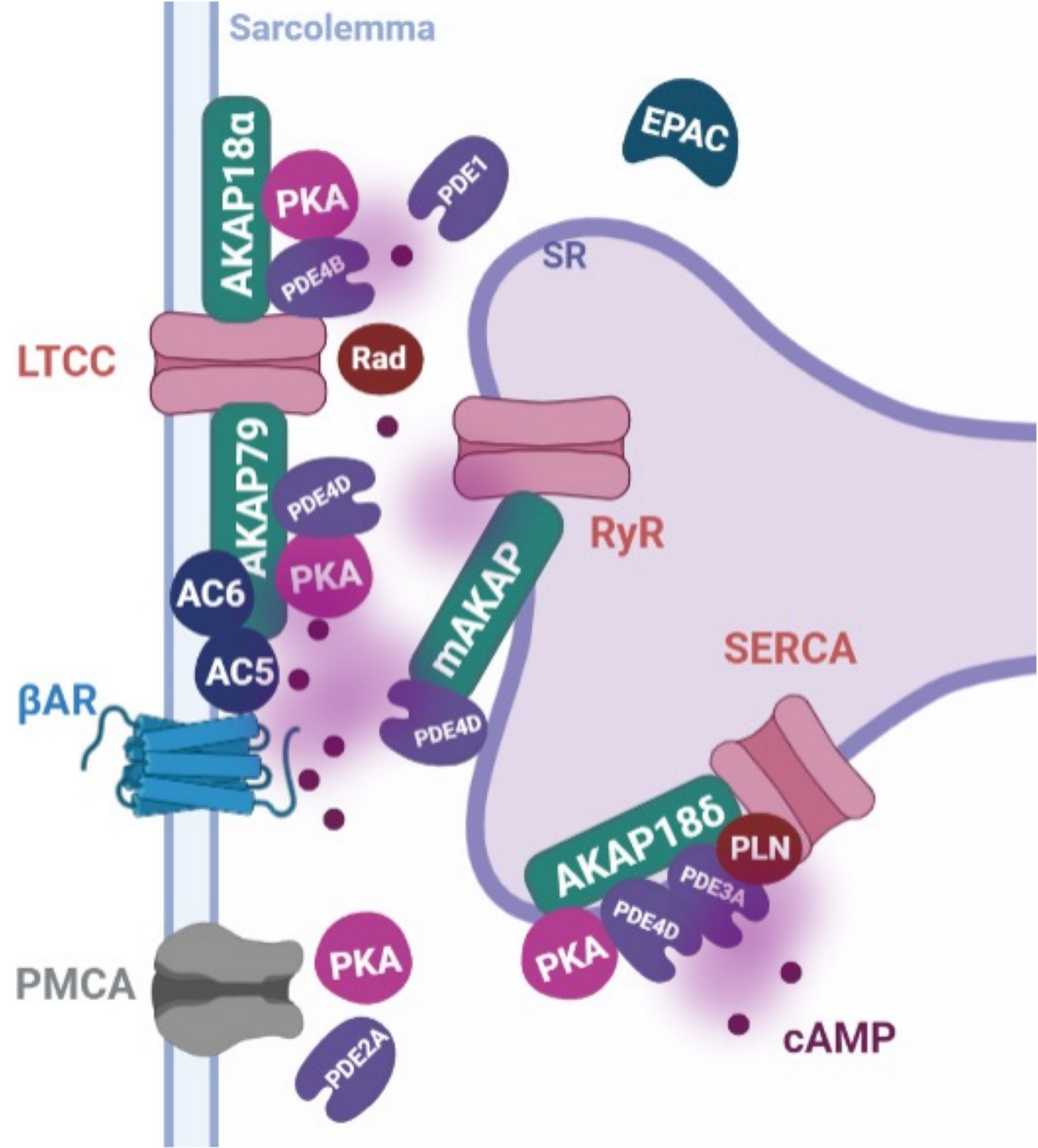
Main cAMP-domains associated with calcium-handling in cardiomyocytes LTCC are anchored by AKAP18α, and cAMP levels near the channel are predominantly controlled by PDE4B, PDE4D, and PDE3. PDE1 appears to be involved in signaling regulation as well. In proximity to LTCCs, AC5 and AC6 produce cAMP. Both cyclases are known to interact with AKAP79. On the membrane, calcium signaling via PMCA channels influences calcium signaling in the cell. On the SR, AKAP18δ anchors SERCA, PLN, PKA, and PDE3A with PDE4D controlling cAMP signalosome. Calcium release via RyR is also regulated by cAMP. RyR are in proximity to mAKAPβ that also interacts with PDE4D. Abbrreviations: AC, adenylyl cyclase; AKAP: A-kinase anchoring protein; βAR, β adrenergic receptor; EPAC, exchange protein directly activated by cAMP; PDE, phosphodiesterase; PKA, protein kinase A; PLN, phospholamban; PMCA, plasma membrane calcium ATPase; LTCC, L-type calcium channel; RyR, Ryanodine channel; SERCA, SR calcium ATP-ase, SR, sarcoplasmic reticulum.

One of cAMP domains associated with calcium handling includes LTCCs. Upon cAMP production following βAR stimulation, PKA anchored to AKAP18α is activated leading to increased open probability of the LTCC in cardiomyocytes [57–60]. Recently, it has been reported that, rather than via direct phosphorylation of LTCCs, this is achieved via phosphorylation of Rad, a member of the Ras-related GTP-binding protein subfamily, that, in its unphosphorylated state, inhibits LTCC in mouse cardiomyocytes [61–64]. PDE4B and PDE4D appear to be the predominant PDEs that degrade cAMP in proximity to LTCC, at least in rodents [65] (Figure 2). However, additional control of signal specification is provided by GPCRs and AC5 and AC6, which are in close proximity to LTCCs [66]. For example, on β2AR stimulation, regulation of cAMP levels in proximity of LTCC appears to be predominantly dependent on PDE3 followed by PDE4, whereas cAMP generated in response to β1AR is mostly degraded by PDE4, PDE3, and PDE2 [67]. Recently, Muller et al. reported that PDE1 inhibition after βAR stimulation increases CaV1.2 conductance without affecting the phosphorylation of other PKA targets (PLN, TnI, or myosin-binding protein C (MyBPC)) in primary guinea pig myocytes [68]. The precise mechanisms of PDE1-mediated cAMP regulation at LTCC site remain to be elucidated. Besides AKAP18α, AKAP79 and SAP97 are other AKAPs that interact with LTCCs [69,70]. Disruption of the LTCC, PKA, SAP97, and PDE4D8 complex appears to activate LTCC via PKA-dependent phosphorylation due to the reduction in PDE4D8 activity at the signalosome [69]. AKAP79 interaction with AC5/AC6 controls the phosphorylation of ACs, thereby modulating their production of cAMP. Knocking out AKAP79 impairs calcium handling in cardiomyocytes, with a loss of PKA-mediated RyR phosphorylation and SR calcium release [70]. Other cAMP domains localized at the plasmalemma are also important for calcium-handling regulation. For example, canonical transient receptor potential channels (TRPC) expressed in embryonic chick cardiomyocytes form a complex with the LTCC [71]. TRPC3-mediated calcium entry activates PDE1C via CaM, thereby inducing a reduction in cAMP produced by the adenosine A2 receptor–G_αs_ signaling [72].

At the cardiomyocyte SR, SERCA is anchored to AKAP18δ forming a domain with PKA, PDE3A1, PDE4D, SERCA2, PLN, CaM, and CaMKIIδ [73,74]. PKA-mediated phosphorylation of PLN results in the dissociation of PLN from SERCA2, allowing diastolic SR calcium reuptake and myofilament relaxation [60]. Interestingly, cAMP levels raise in the AKAP18δ domain after β1AR but not after β2AR stimulation, and this difference is eliminated after inhibition of PDEs [75], with PDE3 and PDE4 being implicated in the regulation of cAMP at this site [76,77]. Calcium release from SR via RyR is also controlled by cAMP but unlike the SERCA/PLN domain, the regulation of cAMP signaling near RyR in rats is not influenced by PDE2, PDE3, or PDE4 under basal conditions [30]. RyR is anchored by mAKAPβ forming a signalosome including AC5, PDE4D3, and PKA. Only β1AR stimulation induces a significant increase in cAMP near RyR, whereas the increase after β2AR stimulation appears minimal [78–80]. mAKAPβ also forms a signalosome at the nuclear envelope that appears to be involved in cardiac remodeling and response to hypertrophic stimuli [81,82].

#### Diseases

Multiple cardiac disorders associated with mechanical dysfunction, aberrant calcium handling, and arrhythmias show a reorganization of cAMP domains [35,83,84]. Alterations including down-regulation of PDE4A, PDE4B, PDE3A, AKAP79, AC, and β1AR and up-regulation of PDE2 have been reported in HF [85–90]. In a mouse model of mild HF induced by transverse aortic constriction (TAC), PDE2 was found to be redistributed from β1AR (predominant association in healthy hearts) to β2AR, despite global PDE2 expression and activity being unchanged, leading to the hypothesis that such changes may promote the cardiac remodeling found in HF [67].

In another study, PDE4D3 activity was shown to be decreased at the RyR signalosome in failing myocytes, leading to hyperphosphorylation of RyR [91], although the contribution of this mechanism to HF pathogenesis is not clear [92]. In failing myocytes, several PKA targets are hypophosphorylated, including PLN, which accounts for the reduced SERCA2 activity. Studies using a PLN-targeted Fluorescence Resonance Energy Transfer (FRET) sensor demonstrate that even though cytosolic PDE4 activity is reduced in mice models of HF, this activity is retained in the SERCA-PLN cAMP domain, while the contribution of PDE2 to local cAMP control is significantly increased [93]. In addition, the difference between cytosolic- and domain-specific cAMP levels, following βAR stimulation is abolished in disease. Less is known about signalosome changes related to the LTCC domain, besides the observation that LTCC open probability is increased, which could be attributed to alteration of local signaling and phosphorylation events [94]. Whether Rad phosphorylation is affected in HF remains unknown, but its overall expression was found to be decreased in HF, possibly as a compensatory response [95]. Alteration of calcium handling is thought to be detrimental not only for the increased likelihood of arrhythmias but also for the involvement of calcium in the activation of the CaN–NFATc (calcineurin - nuclear factor of activated T cells) pathway. CaN interacts with the mAKAPβ signalosome resulting in NFATc dephosphorylation and translocation into the nucleus, leading to cardiac hypertrophy [96].

#### Potential for drug targeting

Several approaches have been tested in order to restabilize cAMP control of calcium handling in the cells, including attempts to disrupt the interaction of AKAPs with individual binding partners. The AKAP18α-LTCC interaction disrupting peptide AKAP15LZ (38–54) was found to abolish PKA-mediated LTCC phosphorylation [57,97]. So far, no therapeutic use has been suggested for the peptide, but the authors highlight its usability for precise targeting of PKA for regulation of L-type calcium currents [57]. A peptide named ‘PLN peptide’ disrupts the interaction between AKAP18δ and PLN [77]. Although this would be of limited use in HF, as PLN phosphorylation is down-regulated in this condition, physically displacing PLN from SERCA2 might be beneficial in postinfarction, to limit the effects of chronic adrenergic signaling [98].

PDEs have also been considered as potential therapeutic targets in heart disease. For example, the PDE1 inhibitor ITI-214 (lenrispodun) demonstrates a positive inotropic effect and is undergoing phase II trials for the treatment of HF [99]. Due to the localized effects of PDE1 inhibition, targeting PDE1 could provide a novel positive inotropic therapy for HF without off-site effects commonly observed with PDE3 inhibitors.

Even though the major focus in the drug-development field targeting PDEs remains the search for isoform-specific inhibitors and activators, a vastly unexplored avenue is the disruption of PDE–AKAP interactions. Displacement or selective activation of PDEs within an individual signalosome would allow higher precision in the therapeutic intervention.

### Fine-tuning of myofilament contraction and relaxation

Besides calcium-related processes, cAMP signaling ensures proper cardiac contraction and relaxation via the regulation of myofilament proteins. PKA-dependent phosphorylation of myofilament proteins tunes calcium sensitivity of troponin and modulates MyBPC activity, increasing the speed of cross-bridge formation [100]. Evidence suggests that cAMP compartments at the myofilament are sufficiently small, so that proteins such as TnI and MyBPC may be under the control of distinct cAMP nanodomains [28]. Dysregulation and reorganization of PDE signaling at the contractile apparatus appears to be implicated in cardiovascular disorders.

#### Domains

**Figure 3:**
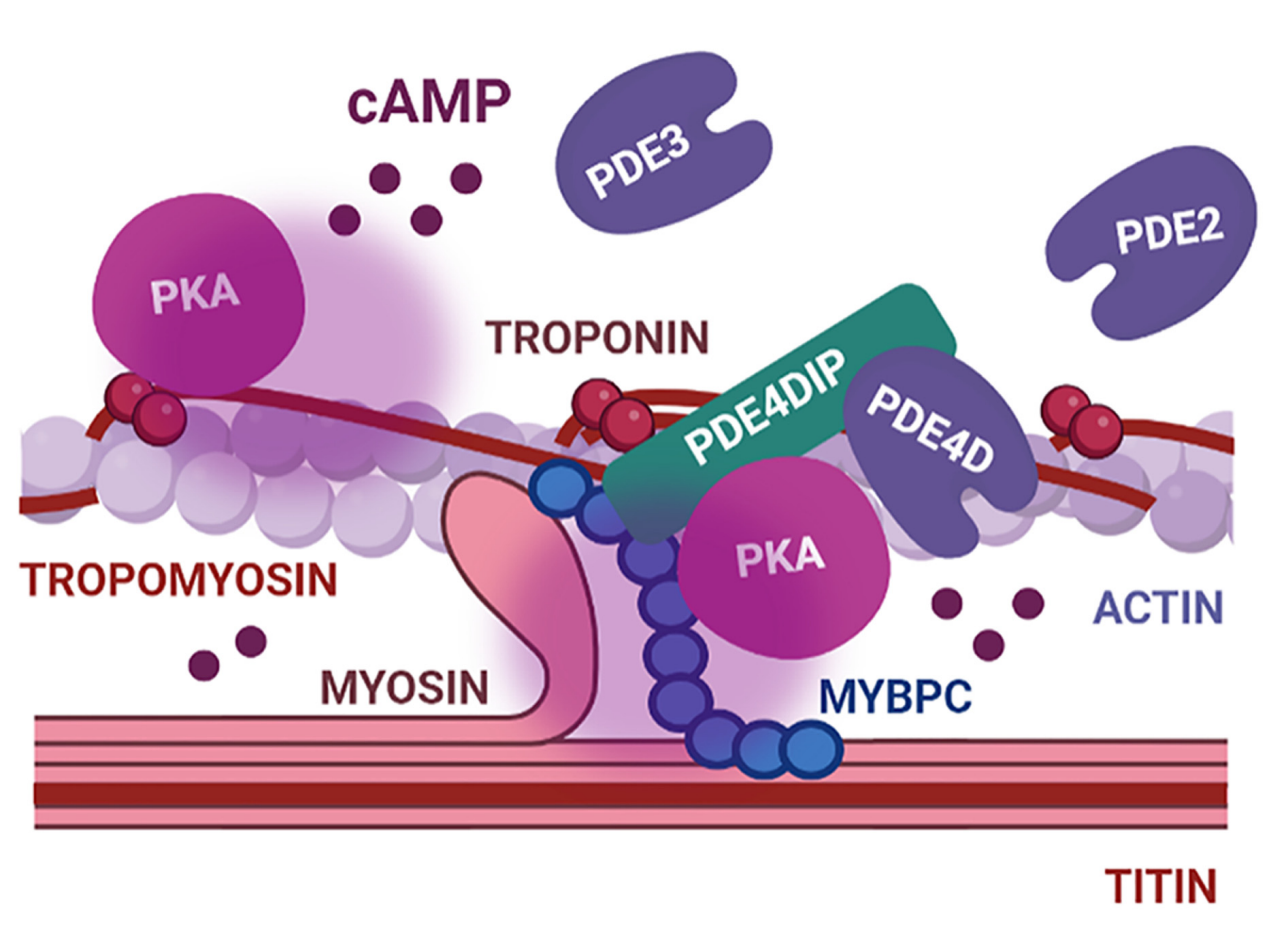
cAMP signaling domains related to myofilament function regulation in cardiomyocytes This nanodomain includes PKA anchored by the AKAPs TnT and PDE4DIP. cAMP signaling at the myofilament is controlled by PDE4, PDE3, and PDE2. Abbreviations: PDE, phosphodiesterase; PDE4DIP, PDE4D-interacting protein; PKA, protein kinase A.

Upon βAR activation, PKA phosphorylates TnI at position S23 and S24 leading to faster relaxation and increased cross-bridge cycling rate as a consequence of reduced sensitivity of the myofilament to calcium [101,102]. The troponin complex consists of TnI, troponin C and troponin T (TnT). The latter acts as an AKAP, tethering PKA to the troponin complex [103]. Interestingly, cAMP increase following βAR stimulation is significantly lower in proximity of TnI than in the cytosol, or at other signalosomes such as AKAP18δ [32], and different PDEs seem to be involved in cAMP compartmentation at the myofilament (Figure 3).

Under βAR stimulation, PDE4 and PDE3 seem to be the main phosphodiesterases controlling cAMP levels near TnI, and PDE2 contributes to a lesser extent in rat hearts [30]. However, in the absence of βAR activation, PDE4 inhibition does not affect sarcomere shortening or TnI phosphorylation suggesting that PDE4 might be recruited to the myofilament following βAR stimulation. Both MyBPC and TnI interact with myomegalin (also called PDE4DIP), an AKAP involved in the control of MyBPC and TnI phosphorylation [104]. The exact localization and constitution of the domain associated with PDE4DIP is unclear, although mutations in PDE4DIP are known to impair cAMP signaling at the myofilament giving rise to arrhythmias [105].

The PDE4D-interacting protein MyBPC is phosphorylated at up to four sites by various kinases, including PKA, and its phosphorylation is down-regulated in failing human hearts [106]. PDEs are important for reducing cAMP levels in the MyBPC-associated nanodomain, and inhibition of PDE2, PDE3, or PDE4 increases MyBPC phosphorylation [30].

Whether PDEs regulate PKA-dependent phosphorylation of titin remains to be fully investigated. This is of importance as titin is essential in the regulation of passive stiffness and its role in other processes such as active tension generation, is emerging [107]. It is reported that PKA-dependent phosphorylation of the titin region N2B causes a decrease in stiffness enhancing diastolic filling during adrenergic stimulation [108]. Mutations in titin are known to cause diseases such as dilated cardiomyopathy (DCM) and HF, but the mechanisms are not fully understood. Titin binds multiple proteins in the myofilaments including α-Synemin, an AKAP that anchors PKA and PP2A to the M-band [109]. Besides PP2A, PP5, and PP1 have been reported to dephosphorylate titin, but only PP5 effects have been shown *in vivo* [110]. Titin size and its multiple interactors as well as multiple phosphorylation sites make studying local cAMP regulation of titin more challenging.

#### Diseases

Disruption of cAMP-dependent regulation of myofilament function is associated with multiple cardiovascular disorders. Reduced phosphorylation of TnI and MyBPC has been reported in human and experimental HF samples, despite total PKA activity following cAMP stimulation being comparable between failing and nonfailing human hearts [106,111,112]. In some animal models, the phosphorylation of TnI appears to be increased rather than decreased [113]. However, in rat hearts, the cAMP signal triggered by βAR stimulation was found to be significantly reduced at the myofilament but preserved at the SR and in the cytosol [32]. In hypertrophic rabbit myocytes, PKA signaling at the myofilaments was reportedly up-regulated inducing contractile dysfunction due to a redistribution of PDEs [114]. It is likely that, in the failing myocyte, rather than just down-regulation or inactivation, PDEs and subsequently PKA and possibly AKAPs may undergo a reorganization and redistribution, which may result in hypo- or hyperphosphorylation of TnI and MyBPC and may be specific to diseases of different etiologies and possibly to different stages of disease.

A123T mutations in PDE4DIP are associated with early-onset atrial fibrillation and heart block, which appears to be induced by impairment of PKA and PDE4D compartmentation, resulting in increased cAMP near the β2AR site and decreased PKA phosphorylation of desmin [105]. The TnT R173W mutation presents with impaired cardiomyocyte contractility due to disrupted interactions between troponin and tropomyosin, and reduced PKA binding to sarcomere domains, causing TnI hypophosphorylation in DCM [115].

Similar to TnI and MyBPC, the phosphorylation profile of titin is also altered in cardiac disorders. In HFpEF and cardiomyopathies (DCM and HCM), cardiomyocytes appear to be stiffer having an increased passive tension, which is thought to depend on hypophosphorylation of titin [116,117]. Currently, little is known about titin-associated cAMP signalosomes and their reorganization in disease.

#### Potential for drug targeting

Myofilament proteins regulated by cAMP via PKA are hypophosphorylated in human HF and cardiomyopathies, in line with findings demonstrating that, despite maintained cAMP levels at the cytosol, cAMP signals triggered by βAR stimulation are significantly reduced at the myofilament [32]. Interestingly, the main PDEs known to regulate cAMP at the myofilament are underexpressed in failing hearts, although changes in their spatial (re)distribution are underexplored highlighting the need for further investigations [89,91]. Currently, there are no pharmacological agents that aim to modulate PKA activity at the myofilament, or the myofilament-associated interactions with AKAPs or PDEs.

### Regulation of cardiac gene expression by cAMP

cAMP is an important regulator of gene expression. cAMP-dependent targets in this context include cAMP response element-binding protein (CREB), NFAT, and glycogen synthase kinase-3β (GSK-3β). Dysregulation of these signaling cascades is associated with the development of hypertrophy and other cardiac disorders. So far, limited therapeutic approaches have been proposed in order to modulate regulation of cAMP-mediated gene expression.

#### Domains

**Figure 4:**
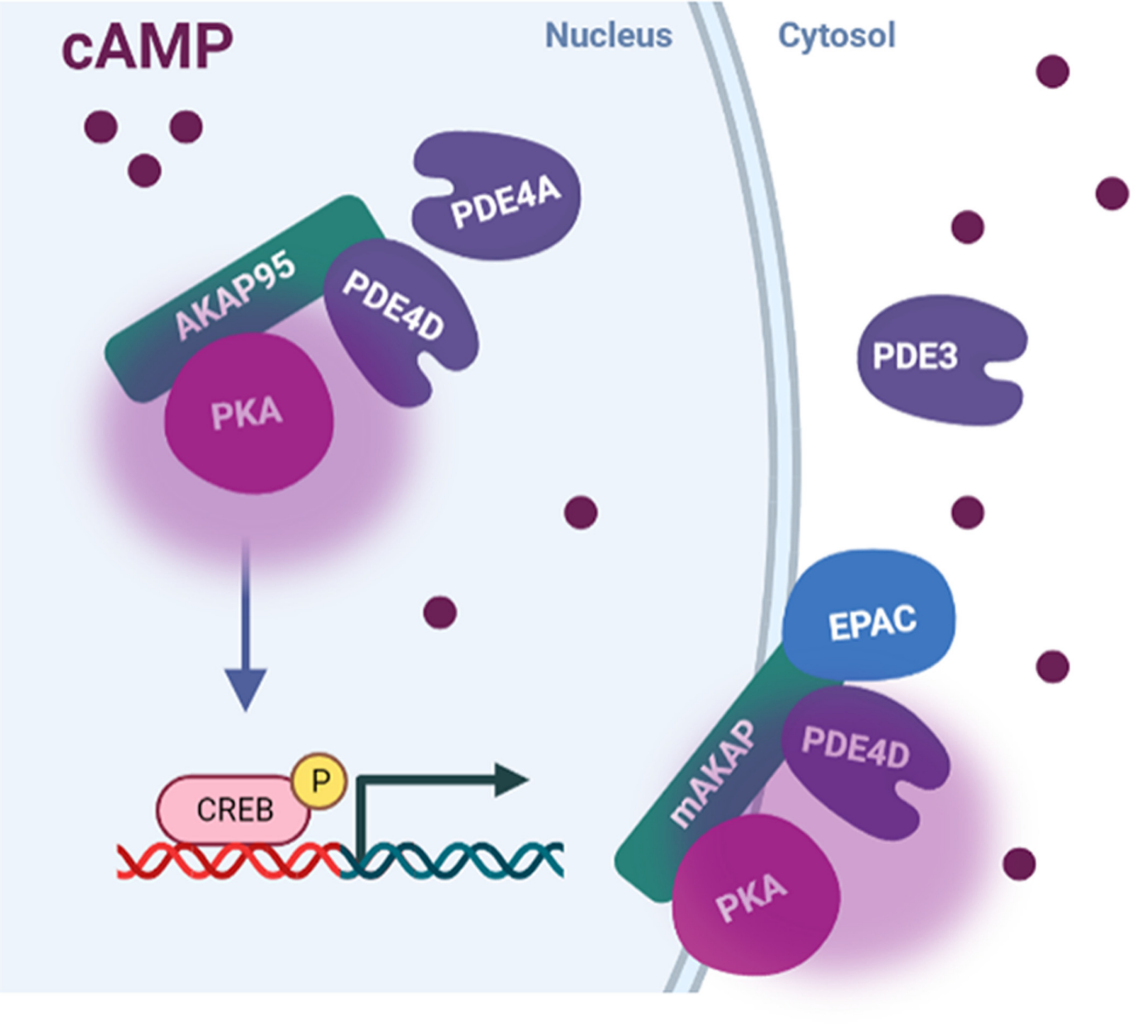
cAMP domains associated with gene expression regulation cAMP in the nucleus is produced by soluble ACs. AKAP95 was found to anchor PKA in the nucleus in HEK293T cells, forming a cAMP domain regulated by PDE4A and PDE4D. cAMP in the nucleus regulates gene expression related to cell hypertrophy, apoptosis, and other processes. On the nuclear membrane, another cAMP domain includes mAKAPβ, EPAC, PKA, and PDE4D. Abbreviations: AKAP, A-kinase anchoring protein; CREB, cAMP response element-binding protein; EPAC, exchange protein directly activated by cAMP; PDE, phosphodiesterase; PKA, protein kinase A.

CREB is a transcription factor involved in the control of the expression of multiple genes including somatostatin, c-fos, genes coding for enzymes involved in gluconeogenesis, neuropeptides, and circadian clocks components, such as PER [118]. Through CREB, cAMP regulates the expression of metabolic, cell cycle, and cell survival pathways. CREB initiates transcription after activation by cAMP response element (CRE), the activity of which is induced by PKA phosphorylation at S133 [119]. CRE can also be phosphorylated by other kinases such as CaMKII [120]. In HEK293 cells, CREB-signaling domain includes AKAP95 (AKAP8), PP2A, PP1, PDE4D5, and PDE4A3 [120–122] (Figure 4). Local cAMP generation within this domain upon βAR activation is thought to be executed by AC10, an isoform that is not activated by G_s_ and is not associated with the plasmalemma. Experimental and simulation studies show that activation of β2AR has a significant effect on CREB and GATA4 activity, which may exacerbate hypertrophic gene expression in HF when the β1/β2 AR ratio decreases [123]. Studies in HEK293T cells suggest that the CREB-associated cAMP domain is not activated by plasmalemma-bound ACs, as cytosolic PDE3 provides a barrier-preventing accumulation in the nucleus of cAMP generated at the cell membrane [121,124].

Another cAMP signalosome involved in the regulation of gene expression is associated with mAKAPβ and involves hypoxia-inducible factor 1α (HIF1α), MEF2, NFATc, and type II HDACs [82,96,125,126]. mAKAPβ facilitates the transcription of pro-hypertrophic genes via cAMP-dependent and cAMP-independent pathways (by activating MAPK effector kinase p90RSK and modulating HIF1α) [127,128]. The signalosome includes PKA, PDE4D3, and Epac1 (Figure 4). Upon cAMP increase, PKA anchored to mAKAPβ phosphorylates PDE4D enhancing its activity and leading to cAMP degradation [129]. mAKAPβ associated ERK5, which is involved in activation of cardiomyocyte hypertrophy [130], suppresses PDE4D3 activity thereby promoting PKA activation. PKA regulates gene expression via the phosphorylation of HDAC, NFAT, and GSK-3β [131,132].

#### Diseases

Multiple studies link cAMP-mediated regulation of gene expression with cardiac disorders. Overexpression of dominant-negative CREB in mice leads to an increase in mortality and mitochondrial dysfunction in mice [133]. Moreover, mice with dominant negative CREB appear to have an impaired contractile response to isoproterenol and cardiac dilatation [134]. CREB has been demonstrated to be important for ion channel function, as in CREB knockout mice action potentials are prolonged [135]. In animal models of HF, phosphorylation of CREB Ser133 appears to be decreased during cardiac remodeling, suggesting that dysregulation of cAMP and PKA control of CREB may contribute to the pathogenesis of this condition [136].

Recent findings show that alterations in G_sα_ expression found in mice after TAC decrease CREB expression and inhibit Bmp10 signaling, leading to cardiac remodeling in HF [137]. Key molecular events contributing to cardiac hypertrophy include MEF2-, CREB-, and NFAT-mediated gene transcription. Mechanistically, upon G_sα_-stimulated cAMP synthesis, PKA activation in the nucleus activates CREB and lifts GSK3β-mediated NFAT repression, which promotes hypertrophy. Due to cAMP and PKA-signaling compartmentation, a cytoplasmic PKA pool appears to play a completely different role: activation of PKA outside the nucleus inhibits hypertrophy through HDAC4/5-mediated MEF2 repression, inactivating NFAT and suppressing Drp1. NFAT5 activation after GSK-3β phosphorylation by PKA is also important for modulating responses to biomechanical stretch, stress, and disease [131,132].

The mAKAPβ domain is also implicated in hypertrophy. mAKAPβ-bound PKCε and PKD activation secondary to EPAC-PLCε–dependent phosphatidylinositol-4-phosphate (PI4P) hydrolysis, leads to phosphorylation, and nuclear export of type IIa HDACs and induction of hypertrophy [138]. PKA activation acts as a cardioprotective antihypertrophic mechanism inhibiting PLCε-dependent PI4P hydrolysis [139].

#### Potential for drug targeting

Different approaches have been explored to modulate cAMP signaling to control cardiac hypertrophy. Reduction in cAMP in the CREB signalosome via PDE4 activation has been suggested as a potential way to reduce hypertrophy. PDE4 activation has also been proposed as a therapeutic approach. The PDE4 activator UCR1C has been shown to attenuate cardiomyocyte hypertrophy in preclinical models, and its positive effects are executed via the regulation of PKA nuclear activity [140]. Other PDE4 long-form activators, such as MR-L2, have been proposed for the treatment of polycystic kidney disease, but their potential benefit in heart diseases remains unclear [141].

Other approaches include the disruption of the interaction between AKAPs and other signaling molecules. For example, disruption of AKAP-Lbc and p38 MAPK interaction appears to protect mice from hypertrophic remodeling in response to TAC [142]. Another useful approach for treating HF-associated cardiac remodeling could be implemented by using the peptide CaNBD that disrupts mAKAPβ-CaN interaction. Such modulation has been reported to reduce hypertrophy in rat cardiomyocytes via modulation of the NFAT pathway [143]. mAKAPβ delocalization via disruption of mAKAPβ-nesprin interaction appears to have a cardio-protective effect mediated by ERK5 inhibition. As mAKAPβ-nesprin anchors PDE4D3 with ERK5, MEK5, and EPAC1, ERK5 activation induces PDE4D3 phosphorylation and inhibition, which in turn enhances PKA/RyR/cytoplasmic calcium/CaN/nuclear NFATc3 pathway [130]. Additional control of ERK5 is provided by EPAC1 that, when activated, inhibits ERK5 activity, and acts as an antihypertrophic agent. Therefore, mAKAPβ-nesprin-disrupting peptides can potentially elicit precisely executed antihypertrophic effects [130].

## Conclusions and future perspectives

Cardiovascular disease continues to exert a substantial burden on the healthcare system. Gaining a better understanding of the local cAMP-signaling organization could be key in developing precise and effective therapies. As evidenced by the previous studies using PDE inhibitors, targeting global intracellular cAMP signals rarely provides the desired effects, highlighting the importance of more precise therapeutic interventions. However, despite exciting new developments in the field, multiple questions remain open and will have to be addressed before cAMP nanodomain signaling can be exploited for therapeutic intervention. Further characterization of cAMP-buffering mechanisms will be essential to fully elucidate the main physiological mechanisms involved in the control of local cAMP levels [36,43]. Mapping cAMP nanodomains, their function, and distinct cAMP control patterns in physiological and pathological settings utilizing multiomics techniques can help dissect and define the details of the intracellular cAMP landscape [144]. The integration of cAMP domain knowledge utilizing computational tools can provide a holistic understanding of cAMP signaling effects in the heart and a framework for *in silico* mechanistic investigations of pathogenic alterations and for simulation of potential therapeutic approaches [145–147]. The combination of new computational and experimental techniques, such as optogenetic modulation of cAMP effectors, has the potential to further expand our understanding of cAMP-signaling organization [148]. Shifting the focus of the investigations from global to domain-specific cAMP signaling will undoubtedly help discover alterations in cAMP signaling in cardiac diseases, which remain to be identified.

## Open Access

Open access for the present article was enabled by the participation of University of Oxford in an all-inclusive *Read & Publish* agreement with Portland Press and the Biochemical Society under a transformative agreement with JISC.

## Abbreviations

βAR: β adrenergic receptor
AC: adenylyl cyclase
AKAP: A-kinase anchoring protein
C: catalytic
cAMP: 3', 5' - cyclic adenosine monophosphate
CaN: calcineurin
CNGC: cyclic nucleotide-gated channel
CRE: cAMP response element
CREB: cAMP response element-binding protein
DCM: dilated cardiomyopathy
EPAC: exchange protein-activated by cAMP
FRET: Fluorescence Resonance Energy Transfer
GPCR: G-protein coupled receptor
GSK-3β: glycogen synthase kinase-3β
HCM: hypertrophic cardiomyopathy
HF: heart failure
HFpEF: HF with preserved ejection fraction
HIF1α: hypoxia-inducible factor 1α
LLPS: liquid–liquid phase separation
LTCC: L-type calcium channel
MyBPC: myosin-binding protein C
NFATc: nuclear factor of activated T cell
PDE: phosphodiesterase
PI4P: phosphatidylinositol-4-phosphate
PKA: protein kinase A
PLN: phospholamban
POPDC: Popeye domain-containing
PP: phosphatase
RIα: PKA regulatory subunit type Iα
RyR: Ryanodine receptor
SERCA: SR calcium ATP-ase
SR: sarcoplasmic reticulum
TAC: transverse aortic constriction
TnI: troponin I
TnT: troponin T
TRPC: transient receptor potential channel
